# Formation of three-dimensional tubular endothelial cell networks under defined serum-free cell culture conditions in human collagen hydrogels

**DOI:** 10.1038/s41598-019-41985-6

**Published:** 2019-04-01

**Authors:** Birgit Andrée, Houda Ichanti, Stefan Kalies, Alexander Heisterkamp, Sarah Strauß, Peter-Maria Vogt, Axel Haverich, Andres Hilfiker

**Affiliations:** 1 Leibniz Research Laboratories for Biotechnology and Artificial Organs (LEBAO), Department of Cardiothoracic, Transplantation and Vascular Surgery, Hannover Medical School, Hannover, Germany; 2 Institute of Quantum Optics, Leibniz University Hannover, Hannover, Germany; 3 Lower Saxony Centre for Biomedical Engineering, Implant Research and Development, Hannover, Germany; 4 Department of Plastic, Asthetic, Hand- and Reconstructive Surgery, Hannover Medical School, Hannover, Germany

## Abstract

Implementation of tubular endothelial cell networks is a prerequisite for 3D tissue engineering of constructs with clinically relevant size as nourishment of cells is challenged by the diffusion limit. *In vitro* generation of 3D networks is often achieved under conditions using serum containing cell culture medium and/or animal derived matrices. Here, 3D endothelial cell networks were generated by using human umbilical vein endothelial cells (HUVECs) in combination with human adipose tissue derived stromal cells (hASCs) employing human collagen I as hydrogel and decellularized porcine small intestinal submucosa as starter matrix. Matrigel/rat tail collagen I hydrogel was used as control. Resulting constructs were cultivated either in serum-free medium or in endothelial growth medium-2 serving as control. Endothelial cell networks were quantified, tested for lumen formation, and interaction of HUVECs and hASCs. Tube diameter was slightly larger in constructs containing human collagen I compared to Matrigel/rat tail collagen I constructs under serum-free conditions. All other network parameters were mostly similar. Thereby, the feasibility of generating 3D endothelial cell networks under serum-free culture conditions in human collagen I as hydrogel was demonstrated. In summary, the presented achievements pave the way for the generation of clinical applicable constructs.

## Introduction

In natural tissues, the nutrition of cells is facilitated by a dense capillary network which is generated during development. A perfusion system is also indispensable for tissue formation *in vitro* to generate tissues of clinically relevant dimensions. Many attempts have been made to reconstruct the microvasculature of native tissues employing a combination of natural or synthetic matrices and endothelial cells (ECs). Different techniques e.g. bioprinting, microfabrication, *in vivo* prevascularization, and self-assembly have been developed for the positioning of matrix and cells^1–4^. Successful EC network formation was described in many approaches, but most studies employed animal derived components like Matrigel or cultivation of the constructs was conducted in medium containing fetal bovine serum (FBS). For envisioned clinical application of such constructs well defined materials as well as serum-free cultivation will be indispensable. The establishment of chemically defined media for cell culture and tissue engineering avoiding serum supplementation is in the focus of research for a couple of years^5^. Supplementation of cell culture medium with FBS has several disadvantages. FBS has an unknown composition and high batch-to batch variability leading to experimental variability and limited inter-laboratory reproducibility^6^. Moreover, ethical concerns about the fetal distress during collection of blood from the unborn calves exist. In addition, a well-defined cell culture medium is easier to manipulate by adding or omitting certain ingredients. A first step towards the direction of serum-free and chemically defined medium for vascular constructs was reported by Huttala *et al*. demonstrating the formation of EC networks of human umbilical vein endothelial cells (HUVECs) in combination with human adipose tissue derived stromal cells (hASCs) in a 2D approach cultivated in vascular stimulation medium (VSM)^7^. The VSM contained DMEM/F12, bovine serum albumin (BSA), ascorbic acid, heparin, hydrocortisone, insulin- transferrin-selenium (ITS), fibroblast growth factor 2 (FGF-2), vascular endothelial growth factor A (VEGF-A), L-glutamine, sodium pyruvate, and 3,3′,5-triiodo-L-thyronine salt. In this approach, no additional matrix was employed as ECs were seeded on a monolayer of hASC. To achieve a 3D construct, hydrogels of different origin are commonly used. Either synthetic materials or natural materials are employed for hydrogels. Natural materials, e.g. rat tail collagen I (rCOL) or Matrigel, originate mainly from animal material due to availability and ease of production. Recently, human collagen I (hCOL) is also available either as extract from extracellular matrix produced by human fibroblasts or as recombinant protein produced in tobacco plants^8,9^. These products are also commercially available. Other available human material like fibrin is challenged by fast degradation through incorporated cells that can only be inhibited by protease inhibitors such as aprotinin^10^. Additionally, fibrin constructs have inferior stability compared to other matrices^10^.

As previously demonstrated by us, the combination of ECs and hASCs in a hydrogel construct containing Matrigel and rCOL leads to EC network formation regardless of the source of ECs^11^. This model is hampered by the usage of animal derived matrices as well as the cultivation in FBS containing medium. Here, we describe the formation of 3D EC networks in serum-free medium adopted from a publication by Huttala *et al*.^7^. In addition, the substitution of Matrigel and rCOL by hCOL from fibroblasts in combination with cultivation under serum-free conditions was investigated.

## Results

### 3D co-culture of HUVCEs with hASCs in serum-free medium enables stable EC network formation

**Figure 1:**
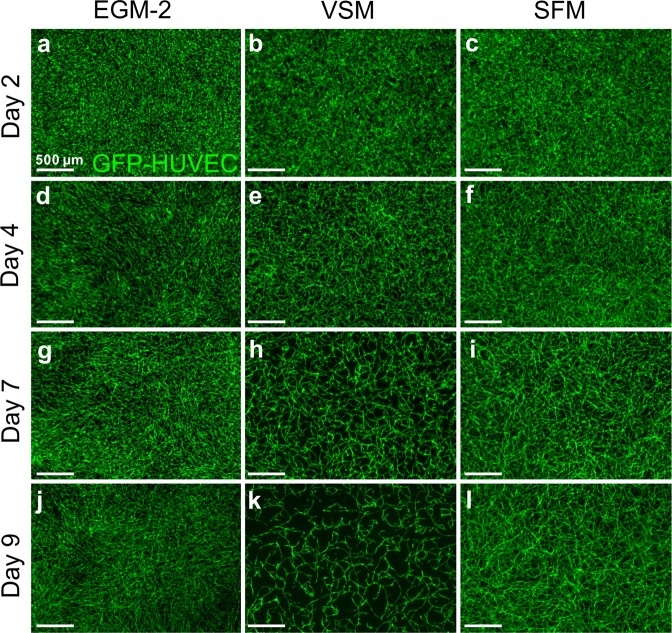
hASCs dependent self-assembly of HUVECs depends on the culture medium. 3D hydrogel constructs containing GFP positive HUVECs and hASCs after 2 days (**a**–**c**), 4 days (**d**–**f**), 7 days (**g**–**i**), and 9 days (**j**–**l**) of cultivation in EGM-2 (**a**,**d**,**g**,**j**), VSM (**b**,**e**,**h**,**k**), and SFM (**c**,**f**,**i**,**l**). Endothelial cell network formation starts between day 2–4 of cultivation under all cultivation conditions. From day 7 onwards endothelial cell networks start to collapse in VSM leading to loss of almost all GFP-HUVEC by day 9. EGM-2: endothelial growth medium-2, VSM: vascular stimulation medium, SFM: serum-free medium, Scale bar: (**a**–**l**): 500 µm

Co-cultures of GFP-HUVECs and hASCs in Matrigel and rCOL containing constructs were cultivated either in EGM-2, serum-free medium (SFM) or vascular stimulation medium (VSM). Formation of EC networks visible by GFP expression of HUVECs was followed over the course of the days (Fig. 1). Constructs cultivated in EGM-2 started to form stable networks from day 3–4 of cultivation onwards as previously described by us^11^ (Fig. 1a,d,g,j). In contrast, cultivation in VSM resulted in a collapse of the initially formed EC network by day 9 of cultivation (Fig. 1b,e,h,k) leading to a complete loss of ECs till the end of the experiment (data not shown). A slight modification of the VSM published by Huttala *et al*.^7^ by removal of sodium pyruvate and T3 from the medium rescued the EC network formation (Fig. 1c,f,i,l). Constructs cultivated in SFM exhibited EC network formation from day 2–3 onwards resulting in a stable, filigree network of GFP-HUVECs.

### Dynamics of EC network formation in SFM

A time-lapse movie was recorded from constructs containing GFP-HUVECs and hASCs after 24 hours of consolidation till day 8 of cultivation (Supplemental Movie 1). Initial signs of organization of GFP-HUVECs were visible after 48 hours of cultivation. Rapid formation of a 3D EC network was observed resulting in stable cords by day 4–5 of cultivation. Although the overall structure of the network was unchanged, dynamic movement of GFP-HUVECs along the cords was visible during the observation period. After formation of the EC network no visible single GFP-HUVECs were apparent indicating that the majority of GFP-HUVECs was integrated into the network.

### hASCs interact with HUVECs under serum-free conditions

**Figure 2:**
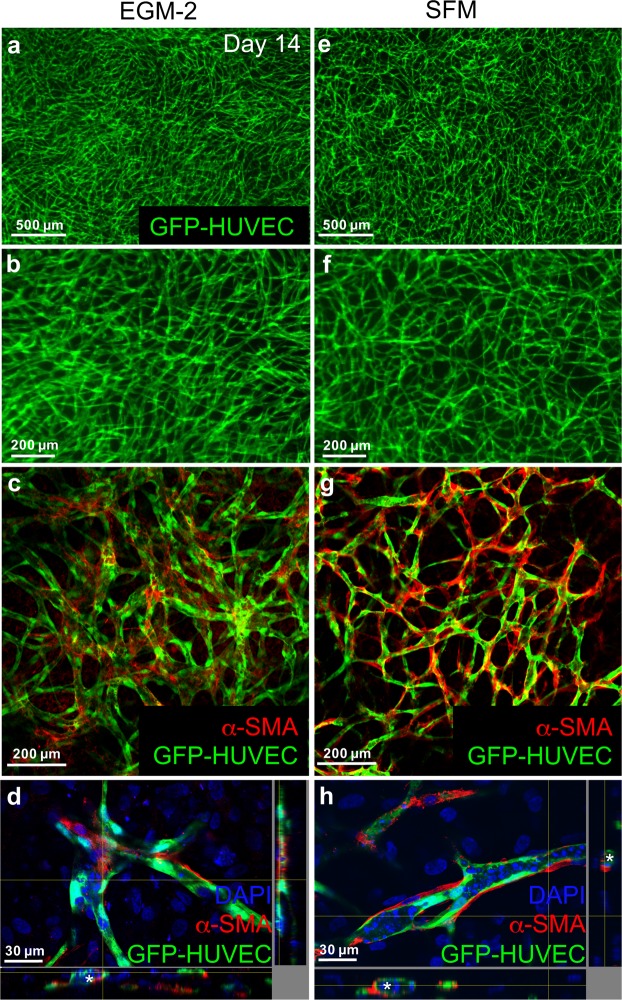
hASCs interact with HUVEC network. 3D hydrogel constructs containing GFP positive HUVECs and hASCs after 14 days of cultivation in EGM-2 (**a**–**d**) or SFM (**e**–**h**). (**a**,**b**,**e**,**f**) Images of GFP-HUVEC network in low and high magnification after 14 days of cultivation. (**c**,**g**) Confocal laser scanning image of α-SMA staining of 3D hydrogel constructs after 14 days of cultivation containing GFP positive HUVECs and hASCs. Cultivation in SFM (**g**) seems to lead to a more extensive coverage of EC cords with α-SMA positive cells compared to cultivation in EGM-2 (**c**). (**d**,**h**) Virtual stacks of GFP-HUVECs co-cultured with hASCs and stained for α-SMA. Nuclei were counterstained with DAPI. α-SMA positive cells are in physical contact with GFP-HUVECs after cultivation in SFM (**h**) or EGM-2 (**d**). Lumen are indicated with asterisks. EGM-2: endothelial growth medium-2, SFM: serum-free medium, Scale bar: (**a**,**e**) 500 µm (**b**,**c**,**f**,**g**) 200 µm, (**d**,**h**) 30 µm.

To compare the interaction of hASCs and HUVECs under different cultivation conditions, constructs were cultivated for 14 days (Fig. 2a,b,e,f) and stained against α-smooth muscle actin (α-SMA) and analyzed by confocal microscopy (Fig. 2c,d,g,h). Under both conditions α-SMA positive cells aligned along the EC cords (Fig. 2c,g) and seemed to be in physical contact with ECs (Fig. 2d,h). In addition, the images from confocal microscopy give the impression that cords in constructs cultivated in SFM seem to be thinner than cords formed in constructs cultivated in EGM-2 (Fig. 2c,g). Moreover, the coverage of the EC network by α-SMA positive cells seems to be more extensive under serum-free conditions compared to FBS containing medium.

### Serum-free cultivation leads to lumenized networks

**Figure 3:**
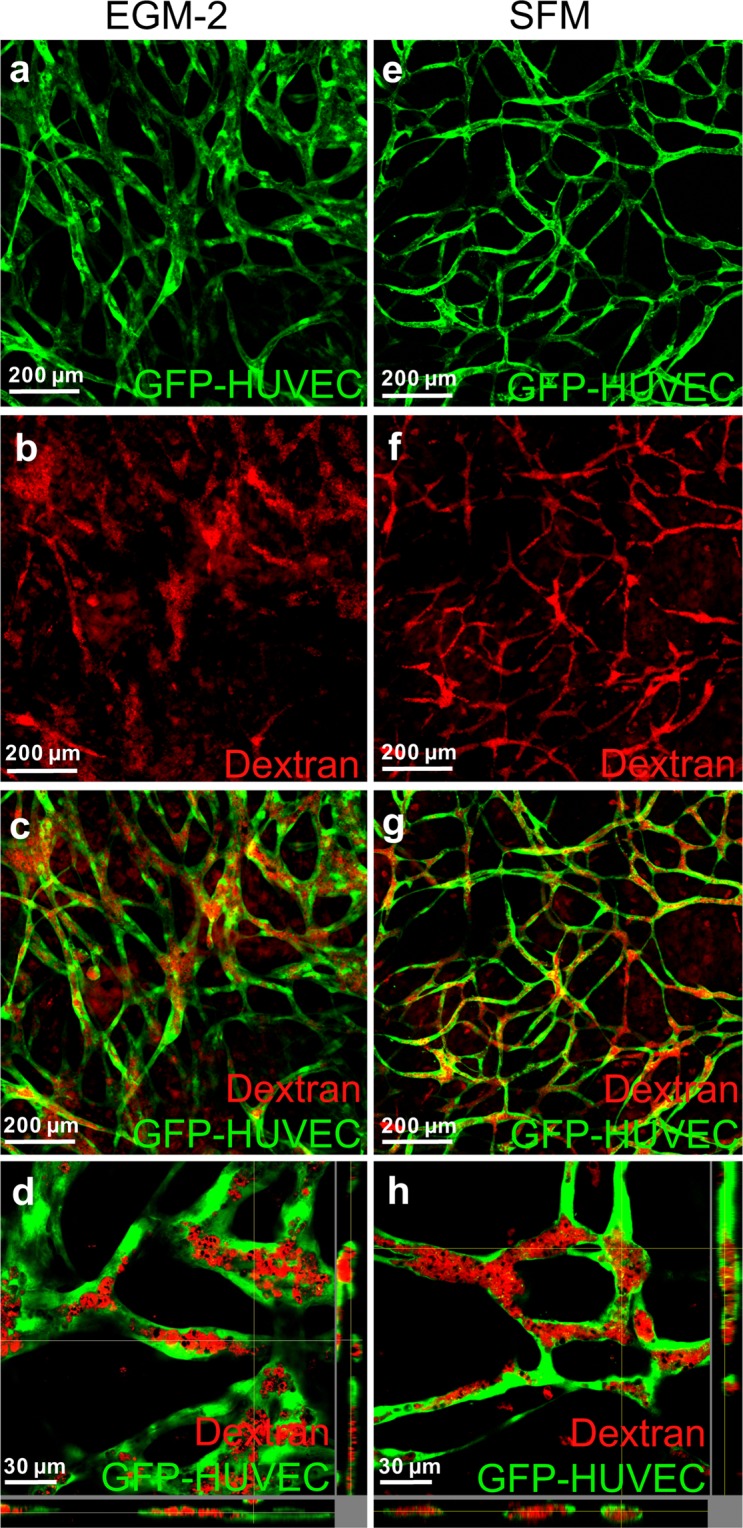
Endothelial cell network possess hollow structures when cultured in EGM-2 and SFM. 3D hydrogel constructs after 14 days of cultivation in EGM-2 (**a**–**d**) or SFM (**e**–**h**) containing GFP positive HUVECs and hASCs after incubation with Texas red-labeled dextran. (**a**,**e**) Images of GFP-Fluorescence. (**b**,**f**) Images of Texas red fluorescence. (**c**,**g**) Overlay of GFP and Texas red. (**d**,**h**) Virtual stacks of HUVECs co-cultured with hASCs and incubated with Texas red-labeled Dextran. EGM-2: endothelial growth medium-2, SFM: serum-free medium, Scale bar: (**a**–**c**,**e**–**g**) 200 µm, (**d**,**h**) 30 µm.

To test for lumen formation, constructs were incubated with Texas red-labeled dextran. In all constructs incorporation of dextran was visible (Fig. 3a–h). Infiltration of Texas red-labeled dextran into the lumen of hollow structures formed by GFP-HUVECs was demonstrated by confocal laser scanning microscopy (Fig. 3d,h). As indicated before, this assay underscores the smaller cord diameter under serum-free conditions compared to FBS containing medium. In addition, the connectivity of the lumenized network seems to be better under serum-free conditions compared to EGM-2 (Fig. 3b,f).

### Human collagen enables EC network formation under serum-free conditions

**Figure 4:**
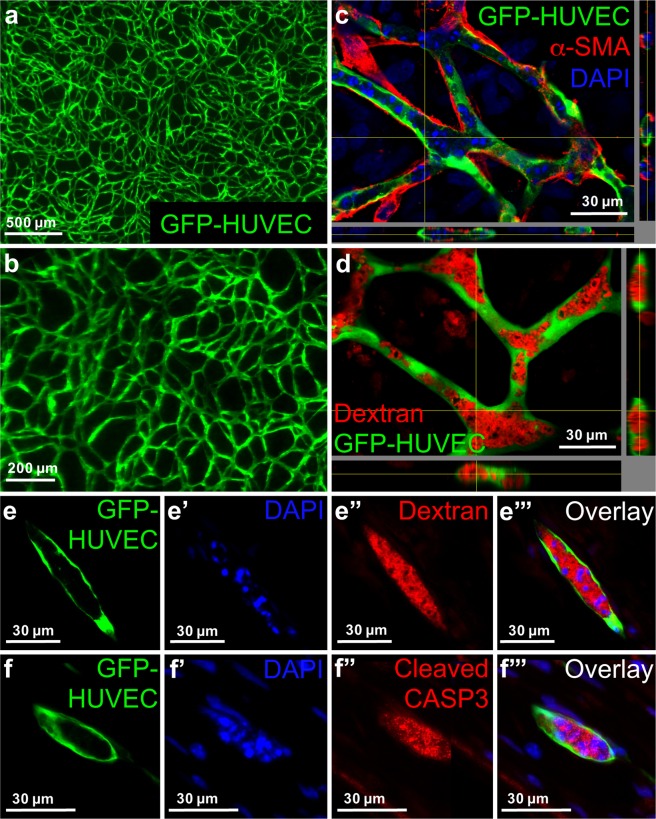
Substitution of Matrigel/rCOL by hCOL from fibroblasts leads to lumenized EC network formation with involvement of apoptosis. (**a**,**b**) 3D hydrogel constructs containing GFP positive HUVECs and hASCs and hCOL after 14 days of cultivation in SFM. (**c**) Virtual stacks of GFP-HUVECs co-cultured with hASCs in hCOL for 14 days and stained for α-SMA. Nuclei were counterstained with DAPI. α-SMA positive cells are in physical contact with GFP-HUVECs. (**d**) Virtual stacks of HUVECs co-cultured with hASCs and incubated with Texas red-labeled Dextran. (**e**–**e**”’) Images of cryo-sections from constructs containing GFP-HUVECs, hASCs and hCOL. Constructs were cultivated for 14 days in SFM and incubated with Texas red-labeled dextran. Cryo-sections were stained with DAPI. (**e**) GFP-HUVEC, (**e**’) DAPI, (**e**”) Texas red-labeled dextran, (**e**”’) Superimposed image of (**e**,**e**’,**e**”). Intense DAPI staining of fragmented DNA is localized within the lumen filled with Texas red-labeled dextran. (**f**–**f**”’) Images of cryo-sections from constructs containing GFP-HUVECs, hASCs and hCOL. Constructs were cultivated for 14 days in SFM. Cryo-sections were stained for cleaved caspase 3 and DAPI. (**f**) GFP-HUVEC, (**f**’) DAPI, (**f**”) cleaved caspase 3 (CASP3), (**f**”’) superimposed image of (**f**,**f**’,**f**”). Intense DAPI staining of fragmented DNA is localized within the lumen and co-localizes with the signal for cleaved caspase 3. Scale bar: (**a**) 500 µm; (**b**) 200 µm; (**c**–**f**”’) 30 µm.

In a next step, Matrigel and rCOL were substituted by hCOL from fibroblasts (Fig. 4). Constructs were cultivated in SFM for 14 days. Formation of an EC network was observed from day 3 onwards. The network seems to be less dense and filigree than the networks formed in Matrigel and rCOL matrices (Fig. 4a,b). EC cords were enwrapped with α-SMA positive cells comparable to constructs containing Matrigel and rCOL (Fig. 4c). Moreover, the EC network possessed lumen demonstrated by Texas red-labeled dextran infiltration (Fig. 4d). An intense DAPI staining of small structures was noticeable in constructs under all conditions, which seemed to be located in the luminal structures (Figs 2d,h and 4c). In cryo-sections of Texas red-labeled dextran infiltrated constructs (HUVECs, hASCs, hCOL, SFM) the intense DAPI staining indicative of fragmented DNA was localized in the lumen which was surrounded by GFP-HUVECs and filled with Texas red-labeled dextran (Fig. 4e-e”’). As these DAPI positive dots might be indicative for apoptosis, cryo-sections of hCOL based constructs were stained for caspase 3, a marker for apoptosis. A signal for caspase 3 was detected within the hollow structures of the EC network co-localizing with DAPI positive dots (Fig. 4f-f”’).

### Quantification of network parameters by 3D reconstruction reveals only minor differences between EC networks generated in Matrigel/rCOL and hCOL under serumfree conditions

**Figure 5:**
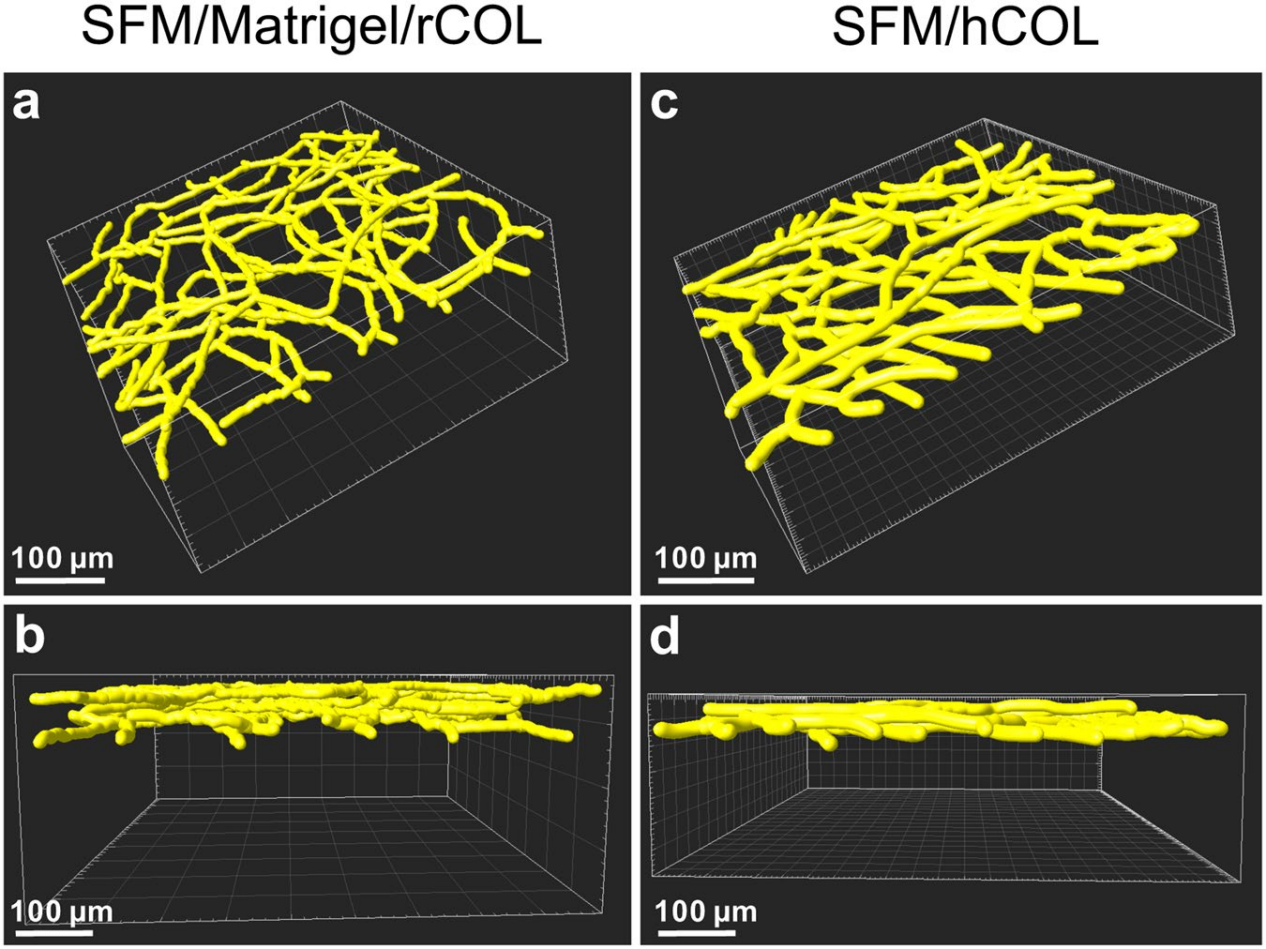
3D reconstruction reveals slight differences in EC network formation. 3D reconstruction of multiphoton images obtained from constructs containing GFP-HUVECs and hASCs and Matrigel/rCOL (**a**,**b**) or hCOL (**c**,**d**) cultivated in SFM for 14 days. (**a**,**c**) 3D reconstruction of EC network employing the calculated mean diameter for each construct. (**b**,**d**) Side view of the 3D reconstruction demonstrating the height of the EC network. Networks generated in hCOL hydrogel are slightly thinner and exhibit a larger mean diameter compared to constructs generated with Matrigel/rCOL. Scale bar: (**a**–**d**) 100 µm.

For the quantification of network parameters constructs containing either Matrigel/rCOL or hCOL were cultivated in SFM for 14 days. Z-stacks of the EC network were obtained by multiphoton imaging. Stacks were processed and the network parameters were calculated with Imaris (Fig. 5, Table 1, Supplemental Movies 2 and 3). From the visual inspection of the reconstruction the general structure of the EC network seems to be similar between the two conditions (Fig. 5a,c). Although, the mean diameter of the EC network in hCOL is larger than in Matrigel/rCOL it does not reach statistical significance but also attributes to the larger network volume in hCOL constructs (Table 1). In Matrigel/rCOL based constructs the number of nodes and segments, as well as the total branching length are larger than in hCOL constructs (Table 1). This is in line with the visual observation that the EC network in Matrigel/rCOL constructs is slightly more filigree than in hCOL constructs.

**Table 1:** Comparison of network parameters.

	SFM/Matrigel/rCOL	SFM/hCOL	Significance
Mean Diameter (µm)	9.3 ± 2.4	12.2 ± 3.3	ns
Number of nodes	113.0 ± 19.3	81.7 ± 4.0	ns
Total branching length (µm)	10747.0 ± 938.9	8357.3 ± 526.6	*
Network area (µm^2^)	(3.2 ± 0.3)*10^5^	(3.2 ± 0.1)*10^5^	ns
Network volume (µm^3^)	(7.7 ± 1.0)*10^5^	(10.4 ± 0.8)*10^5^	*
Number of segments	207.3 ± 34.6	146.3 ± 4.2	*

Constructs containing GFP-HUVECs and hASCs and Matrigel/rCOL or hCOL were cultivated in SFM. After 14 days of cultivation multiphoton images were taken and processed with Imaris. Data is presented as mean ± standard deviation (SD) for the following parameter: mean diameter, number of nodes, total branching length (sum of the lengths of all segments within the 3D network), network area (the sum of areas of all segments within the 3D network), network volume (the sum of volumes of all segments within the 3D network), and number of segments. Data was analyzed with unpaired t-test. (*) p-value ≤ 0.05, ns: not significant.

The height of the resulting EC network is also slightly larger in Matrigel/rCOL constructs (∼75 µm) than in hCOL constructs (∼50–60 µm) (Fig. 5b,d). Supplemental Movies are provided with a rotation of the 3D reconstruction for better visual inspection of the network (Supplemental Movies 2 and 3 for Matrigel/rCOL and hCOL constructs, respectively).

## Discussion

Here, a serum-free medium was adopted from Huttala *et al*.^7^ and optimized for 3D culture of HUVECs and hASCs in a hydrogel construct. A previously established 3D culture model was used to study the influence of serum-free medium on network formation^11^. Employment of serum-free medium enabled the formation of an EC network (Figs 1 and 2), which is covered and in physical contact with α-SMA positive cells (Fig. 2) and whose hollow structures can be infiltrated with dextran (Fig. 3) comparable to the previously established model using EGM-2 for cultivation. These constructs still contain animal derived Matrigel and rCOL. Although animal derived collagens such as rCOL are frequently used matrices in tissue engineering and are easily available, increasing concerns exist about their purity, safety, and immunogenicity. Moreover, the widely used Matrigel is animal derived and consists of a mixture of extracellular proteins that are purified from Engelbreth-Holm-Swarm mouse sarcoma^12^. It contains approximately 60% laminin, 30% collagen IV, and 8% entactin. Additionally, Matrigel includes heparin sulfate proteoglycan and several growth factors such as TGF-beta, epidermal growth factor, fibroblast growth factor, insulin-like growth factor, and tissue plasminogen activator. As these animal-derived matrices are not FDA approved, alternative materials have to be investigated. Here, we have used collagen I prepared from the extracellular matrix secreted by human fibroblasts as a substitute for both, Matrigel and rCOL. The employed human collagen consists of around 97% collagen I and 3% type III collagen. As this collagen is produced by human cells the natural structures and posttranslational modifications should be similar to the native collagen in tissues. Recently, also recombinant human collagens produced in bacteria, yeast, or plants are available but the recombinant expression is challenged due to significant post translational modifications needed for the function of collagens (reviewed in Wang *et al*.^13^). However, recombinant human collagens have been already used in tissue engineering of corneal substitutes^14,15^, skin tissue engineering^16,17^, bone graft substitutes, dentistry, and cartilage reconstruction^17^. In addition, human collagen extracted from abdominal dermis was used successfully for the generation of full-thickness human skin models^18^. This source of human collagen is limited by availability and challenged by biological variation.

In contrast to Matrigel, human collagen produced by fibroblasts probably does not contain growth factors although this has not been investigated to our knowledge so far. The absence of angiogenic growth factors in hCOL could be a disadvantage for network formation compared to Matrigel. However, in our previous research we could demonstrate that growth factors present in the Matrigel are dispensable for endothelial cell network formation when they are cultivated in medium containing a complex mixture of growth factors^11^. Here, constructs containing hCOL were cultivated for 14 days in SFM which contains only FGF-2 and VEGF-A. Despite the limited number of growth factors in the culture medium and probably the absence of growth factors in the matrix, the resulting constructs exhibited similar properties with regard to network formation, coverage and physical contact with α-SMA positive cells, and infiltration with dextran (Fig. 4) compared to Matrigel/rCOL constructs cultivated either in EGM-2 or SFM. Apparently, the amount of growth factors is sufficient to induce and maintain endothelial cell network formation for at least 14 days also in hCOL hydrogels.

Another ingredient of SFM is BSA which has been widely used in the development of serum-free media. BSA is the major protein in FBS and has been identified as a desirable factor for growth of different primary cells and cell lines. Interaction of albumin with the cell with respect to interaction of albumin with ligands and bioactive molecules in the cell culture has an impact on cell proliferation, cell survival, and metabolic activity (reviewed in Francis^19^). Although concerns exist about animal derived components and transmission of related biological contaminants, BSA is used in the production of clinical products such as monoclonal antibodies. Such BSA is isolated from healthy and BSE-free cattle with GMP compliant procedures. Moreover, products that contain BSA have been approved by the FDA for direct use in patients, e.g. BioGlue which contains 45% BSA and is applied as surgical adhesive e.g. in cardiac surgery^20^. In December 2017, the FDA approved PreveLeak surgical sealant which contains BSA as well. Although anti-BSA antibody levels were elevated in patients receiving treatment with BSA containing surgical sealant, no adverse clinical events were reported due to this finding^21^.

With regard to the properties of the generated construct the resulting dimensions are 2.25 cm^2^ with a height of approximately 150 µm (50–100 µm height from SIS, 50–75 µm from hydrogel construct). Depending on the metal frame employed the construct can be as large as 11 cm^2^. The height of the construct can be increased by increasing the number of cells as well as adding other components that hinder the shrinkage of the hydrogel upon gelation (preliminary data). Analysis of the generated network revealed noticeable intense DAPI staining of structures, that were smaller than a cell nuclei and that were located within the lumen (Fig. 4). This is indicative of apoptotic bodies generated while cells undergo programmed cell death. Moreover, a positive staining for the active form of caspase 3 was detected within the lumen underlining the probability of apoptotic processes during lumen formation in the EC network (Fig. 4). To clarify which cells (HUVECs or entrapped hASCs) go into apoptosis further experiments have to be conducted. Actually, a role of apoptosis in lumen formation has been reported for angiogenesis *in vitro*, vasculogenesis in human placenta, and retinal angiogenesis in mice^22–24^. The generated structures are hollow as demonstrated by dextran infiltration. Although, differences in dextran infiltration between the different conditions were visible, the assessment is challenged by the unknown process of dextran infiltration. If dextran is incorporated only by diffusion the differences can be also attributed to the different permeability of the walls of the hollow structures. On the other hand, the infiltration of dextran could be an active process as this assay does not work on fixed constructs (data not shown) and thereby can also be indicative of a different activity of EC under different conditions.

Evaluation of the quality of the generated network with regard to human physiology is challenging as vascularization of the human body is tissue dependent. The average capillary density of human tissue is ∼600 capillaries per mm^3^ ^25^. The value varies considerably in the vascular bed of different organs ranging from 2500–3000 capillaries per mm^3^ in brain, kidney, liver, and myocardium to <100/mm^3^ in bone, fat, and connective tissue^25^. The mean distance between adjacent capillaries is estimated to be ∼40 µm. In the adult human heart the capillary distance was determined with ∼24 µm with slight differences between epimyocardium and endomyocardium^26,27^. In the final constructs containing hCOL and being cultivated in SFM the distance between cords varies from 20 to 200 µm resembling approximately the range in native tissue. The mean diameter of EC cords in the constructs is ∼12 µm ranging from 5 µm to 20 µm. With these values the generated cords resemble large capillaries (having a diameter up to 8 µm) or small arterioles (having a diameter of ∼30 µm)^28^. With these parameters similar to native tissue the generated EC network has the potential to nourish envisioned target cells. The long-term goal is the generation of a perfusable starter matrix by combining a central tubing with the here described EC network. The central tubing should provide the supply of nutrients for the construct by perfusion with medium *in vitro* as well as the option for anastomosis to the host circulatory system and direct perfusion with blood upon implantation. The starter matrix can be employed to generate different tissue types by adding layers of target cells e.g. cardiomyocytes for the generation of cardiac tissue.

In summary, we have demonstrated the successful generation of EC networks under serum-free conditions in a defined medium and employing a defined human matrix implying that the generated network depends heavily on cell intrinsic factors. This achievement offers the ability to engineer animal component free constructs leading to more reproducibility of *in vitro* experiments and paving the way for clinical applications. Challenges ahead include the integration of tissue specific cells, e.g. cardiomyocytes, islet cells, hepatocytes, adipocytes, into the construct for generation of functional tissue for tissue replacement and repair. Moreover, anastomosis to the host circulatory system as well as perfusion of the vascularized tissue have to be established.

## Materials and Methods

### Ethics statement

Patient material was processed following approval of the Ethics Committee at Hannover Medical School (file reference 3475-2017) and after obtaining written informed consent from the patients. All tissues were used anonymously for this study.

All experiments were performed in accordance with relevant guidelines and regulations.

### Animal care

This study was approved by the Institutional Review Board and the local Animal Protection Committee, and was conducted according to local government regulations (#10/0214; #11/0458) and Committee protocols of Hannover Medical School and the Research Advisory Committee. All animals received humane care in compliance with the European Convention on Animal Care. All experiments were performed in accordance with relevant guidelines and regulations.

### Preparation of small intestinal submucosa (SIS)

The preparation of decellularized small intestinal submucosa (SIS) was performed as previously described^11,29,30^. In brief, porcine small intestinal segments were isolated from German landrace pigs (18–25 kg) and stored in undiluted Braunol (7.5% povidone-iodine solution in water, B. Braun) at 4 °C. *Tunica mucosa* and *tunica serosa* of intestinal segments were mechanically removed, followed by a chemical decellularization in 1% Triton X-100 in 10 mM TRIS, pH 7.5 under continuous shaking (90 rpm) at room temperature for 24 h. Afterwards, SIS was washed with distilled water for 24 h under continuous shaking, followed by washing with phosphate buffered saline (PBS) supplemented with 1 g/L Vancomycin, 100 mg/L Gentamicin, and 2.5 mg/L Amphotericin B under continuous shaking for 10 days at room temperature with daily change of washing buffer. Finally, SIS was sterilized by 150 Gy gamma-ray irradiation and stored in PBS at 4 °C until further use for a maximum of 6 months. Before use, SIS was cut open along the longitudinal axis, fixed in a metal frame with the submucosal side facing up and covered with culture medium.

### Human umbilical vein endothelial cells (HUVECs)

HUVECs obtained from pooled donors were purchased from Lonza. Cells were cultured in Endothelial Growth Medium 2 (EGM-2, Lonza). Passages 4–6 were used for all experiments.

### Isolation of adult human adipose tissue derived stromal cells (hASCs)

Human adult adipose tissue-derived stromal cells (hASCs) were isolated from human tissues that were obtained from patients undergoing abdominoplasty. Isolation was performed according to a previously published protocol with minor modifications^31^. In brief, fat and connective tissue were minced, followed by addition of 10 mL Collagenase type II solution (760 U/mL, Worthington) per 20 mL of tissue and incubation for 1 hour at 37 °C under shaking. Fatty supernatants were collected and washed with PBS. After centrifugation, the cell pellet was washed with PBS, resuspended and cells were cultivated in EGM-2. The medium was changed after 48 hours followed by a medium change every other day. Cells from passages 2–4 were used for the experiment.

### Preparation of lentiviral supernatants and cell transduction

VSV.G-pseudotyped lentivirus particles were generated as described previously^30^. Briefly, particles were produced in 293 T cells by calcium phosphate co-transfection of the self-inactivating lentivirus plasmid pHR′-SIN-SEW (for eGFP expression)^32^, together with the multi-deleted pCMV-DR8.91 packaging plasmid and the pMD.G envelope plasmid^33^. Virus supernatant, collected at 36 and 48 h after transfection, was cleared by centrifugation and filtered through 0.45 µm-pore filter. Lentivirus particles were concentrated and resuspended in serum-free X-VIVO10 medium (Lonza) and stored in aliquots at −80 °C until use. Virus titer was determined by infection of K562 cells with 1:2 serial dilution of concentrated virus stock and FACS analysis 72 h post-transduction to assess the number of GFP-positive cells. Concentrated lentivirus stock was used to infect target cells. After 48 hours of incubation, the cells were washed with PBS and fresh medium was added.

### 3D hydrogel construct generation

The preparation of Matrigel/rCOL based hydrogel constructs was conducted as previously described^11,29,30^. In brief, 19.1 vol% 3D culture matrix collagen type I from rat tails (Trevigen), 4.6 vol% double distilled H_2_O, 24.3 vol% gel medium, and 10.3 vol% Matrigel^TM^ (Basement Membrane Matrix, BD) were mixed and neutralized with 4.1 vol% 0.4 M NaOH. The cell mixture resuspended in 37.5 vol% of the respective medium was added and mixed with the hydrogel. hCOL based hydrogel constructs were made from collagen solution from human fibroblast (Sigma). The following components were mixed: 59.7 vol% of collagen, 4.4 vol% water, 23.3 vol% gel medium, 6.3 vol% 0.4 M NaOH, and 6.3 vol% of cell suspension in the respective medium. The resulting hydrogels contained 1.8 to 1.9 mg/mL matrix proteins. In hydrogels containing Matrigel/rCOL the final ratio of collagen IV: laminin: collagen I is calculated as 1:2:3.

The gel medium is composed of 98% 2x DMEM, 2 mM L-Glutamine, 100 U/mL penicillin, 100 µg/mL streptomycin. Double concentrated DMEM was made from 1.348 g DMEM powder (Gibco, Invitrogen), and 0.037 g NaHCO_3_ dissolved in 30 mL water. The hydrogel solution containing cells was cast onto SIS fixed in a custom-made metal frame (1.5 cm × 1.5 cm). The final cell density was 13 × 10^6^/mL with a ratio of HUVECs to hASCs of 1:1.75. After 1 hour of incubation in a humidified incubator at 37 °C and an atmosphere with 5% CO_2_, solidified hydrogel constructs were covered with the respective medium. The medium was changed every 2–3 days and ECs network assembling was documented using a Stereo Discovery.V8 microscope equipped with an AxioCam camera and a HXP lamp.

### Medium composition

Constructs were either cultivated in EGM-2 (Lonza), vascular stimulation medium (VSM)^7^, or serum-free medium (SFM, modified from Huttala *et al*.^7^). VSM is composed of DMEM/F12 as basal medium supplemented with 10 ng/mL VEGF-A, 1 ng/mL FGF-2, 0.1% ITS, 1.28 mM L-glutamine, 1% BSA, 2.8 mM NaP (sodium pyruvate), 100 IU/ml P/0.1 mg/ml S, 0.1 nM T3 (3,3′,5-triiodo-L-thyronine sodium salt), 100 µg/mL ascorbic acid, 0.2 µg/mL hydrocortisone. Heparin sodium salt was omitted from the medium as Huttala *et al*. published that it is dispensable^7^ and antibiotics (0.1 mg/mL gentamicin, 100 U/mL penicillin, and 100 µg/mL streptomycin) were added. The SFM used in our study is composed of M199 as basal medium supplemented with 10 ng/mL VEGF-A, 10 ng/mL FGF-2, 0.1% ITS, 2 mM L-glutamine, 1% BSA, 50 µg/mL ascorbic acid, 0.2 µg/mL hydrocortisone, 0.1 mg/mL gentamicin, 100 U/mL penicillin, and 100 µg/mL streptomycin.

### Quantification of network characteristics

Image acquisition of the EC network formed by GFP-HUVECs was performed using a multiphoton microscopy setup employing a Chameleon Ultra II laser system running at 790 nm or 850 nm (Coherent Inc.), a Thorlabs MPM 200 multiphoton microscope body (Thorlabs GmbH, Germany), and an Olympus XLPlan N objective (25x, NA 1.05). Z-stack images with a z-step size of 1 µm were taken at 3 random regions of each construct. Imaris software (Bitplane) was utilized for the 3D reconstruction of the network from these images. The FilamentTracer modul was employed for the 3D quantification of the EC network using an AutoPath algorithm. Data is presented as mean ± SD for the following parameter: mean diameter, number of nodes, total branching length (sum of the lengths of all segments within the 3D network), network area (the sum of areas of all segments within the 3D network), network volume (the sum of volumes of all segments within the 3D network), and number of segments. Data was analyzed with unpaired t-test. A p-value ≤0.05 is determined as significant.

### Immunofluorescence staining

Immunofluorescence staining was performed according to standard protocols and is described below. 3D constructs were fixed with 4% paraformaldehyde in PBS at room temperature for 30 min followed by 3 washing steps using PBS. Constructs were cut out of metal frames and placed on glass slides or embedded in Tissue-Tek O.C.T. compound, shock frozen in liquid N_2_, and sectioned. Specimens (either whole constructs or cryo-sections) were permeabilized with 0.3% Triton X-100 in PBS for 1 hour at room temperature. Blocking was performed with 2% donkey serum in PBS for 20 minutes at room temperature followed by overnight incubation with primary antibody α-smooth muscle actin (α-SMA) (DAKO, 1:400) or cleaved caspase-3 (CASP3) (Cell Signaling Technology, 1:200) at 4 °C. On the next day, the samples were washed three times with PBS and incubated with Cy3-conjugated donkey anti-mouse antibody or Cy3- conjugated donkey anti-rabbit antibody (both Jackson Research, 1:300) for 2 hours at room temperature, respectively. Samples were washed three times with PBS. Nuclei were counterstained with 4′,6-diamidino-2-phenylindole (DAPI) (0.7 μg/mL in PBS) for 10 minutes at room temperature. After additional washing steps with PBS, samples were covered with fluorescent mounting medium and glass coverslips. Specimens were photographed the next day using an Oberserver.A1 microscope or an Olympus FluoView 1000 confocal laser scanning microscope. Virtual stacks were created with ImageJ.

### Lumen formation analysis using Texas red-labeled Dextran

In order to investigate whether the lumen of the assembled EC networks was free of matrix, whole living 3D constructs were incubated with Texas red-labeled Dextran (D1863, 10,000 MW, ThermoFisher Scientific) as described^34^. In detail, 0.5 mg/mL Dextran in EGM-2 or SFM, respectively, was used to incubate 3D hydrogel constructs at 37 °C and 5% CO_2_ overnight followed by 4% PFA fixation at room temperature for 30 min and two subsequent washing steps with PBS. Photographs were taken with the Stereo Discovery.V8 microscope or an Olympus FluoView 1000 confocal laser scanning microscope. Virtual stacks were created with ImageJ.

### Time-lapse microscopy to investigate dynamics of EC networks

To investigate how EC networks were formed and how they arrange over time, time-lapse microscopy was performed using a lumascope microscope (etaluma) equipped with a 488 nm LED light source. Using the Lumaview620 software, a photo was captured every 20 minutes at 37 °C in a standard incubator. Individual images were converted into a movie using ImageJ software. Time-lapse observation was started 24 hours after casting the construct to enable the initial polymerization and consolidation of the construct.

## Supplementary information

Supplemental Movie 1

Supplemental movie 2

Supplemental movie 3

Supplemental information

## Data Availability

The datasets generated during and/or analyzed during the current study are available from the corresponding author on reasonable request.
