# Supplementary material for: Formation of three-dimensional tubular endothelial cell networks under defined serum-free cell culture conditions in human collagen hydrogels
Source: Sci Rep. 2019 Apr 1;9:5437. doi: 10.1038/s41598-019-41985-6 (PMC6443732; doi:10.1038/s41598-019-41985-6)
Supplement: Supplementary file 4 — Supplemental information [file 41598_2019_41985_MOESM4_ESM.pdf]

Formation of three-dimensional tubular endothelial cell networks under defined serum-free cell culture conditions in human collagen hydrogels

Birgit Andrée[a,1], Houda Ichanti[a,1], Stefan Kalies[b,c], Alexander Heisterkamp[b,c], Sarah Strauß[d], Peter-Maria Vogt[d], Axel Haverich[a], Andres Hilfiker[a*]

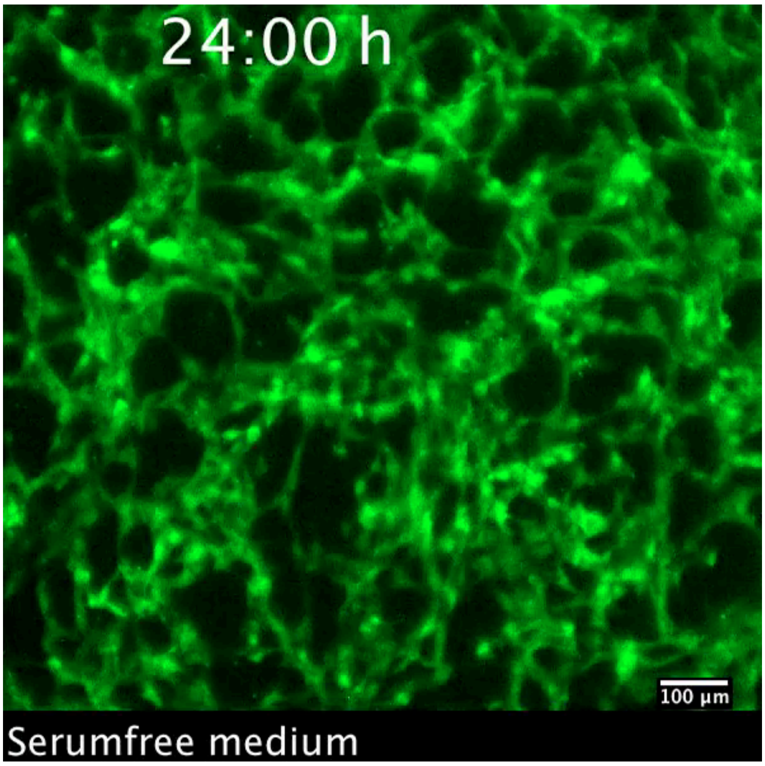

Supplemental movie 1:
Network formation of GFP-HUVECs starts from day 3 onwards resulting in a stable and stationary spatial arrangement.
Time-lapse movie of network formation of GFP-HUVECs in 3D constructs containing hASCs, Matrigel/rCOL, and cultivation in SFM. Time-lapse was started 24 hours after construct generation and recorded for 7 days. Medium was exchanged every 3 days (jumps in movie). Initial network formation started at day 2-3. Re-arrangement of the initial network was very limited and GFP-HUVEC movement was only observed along the existing cords. Scale bar: 200 µm

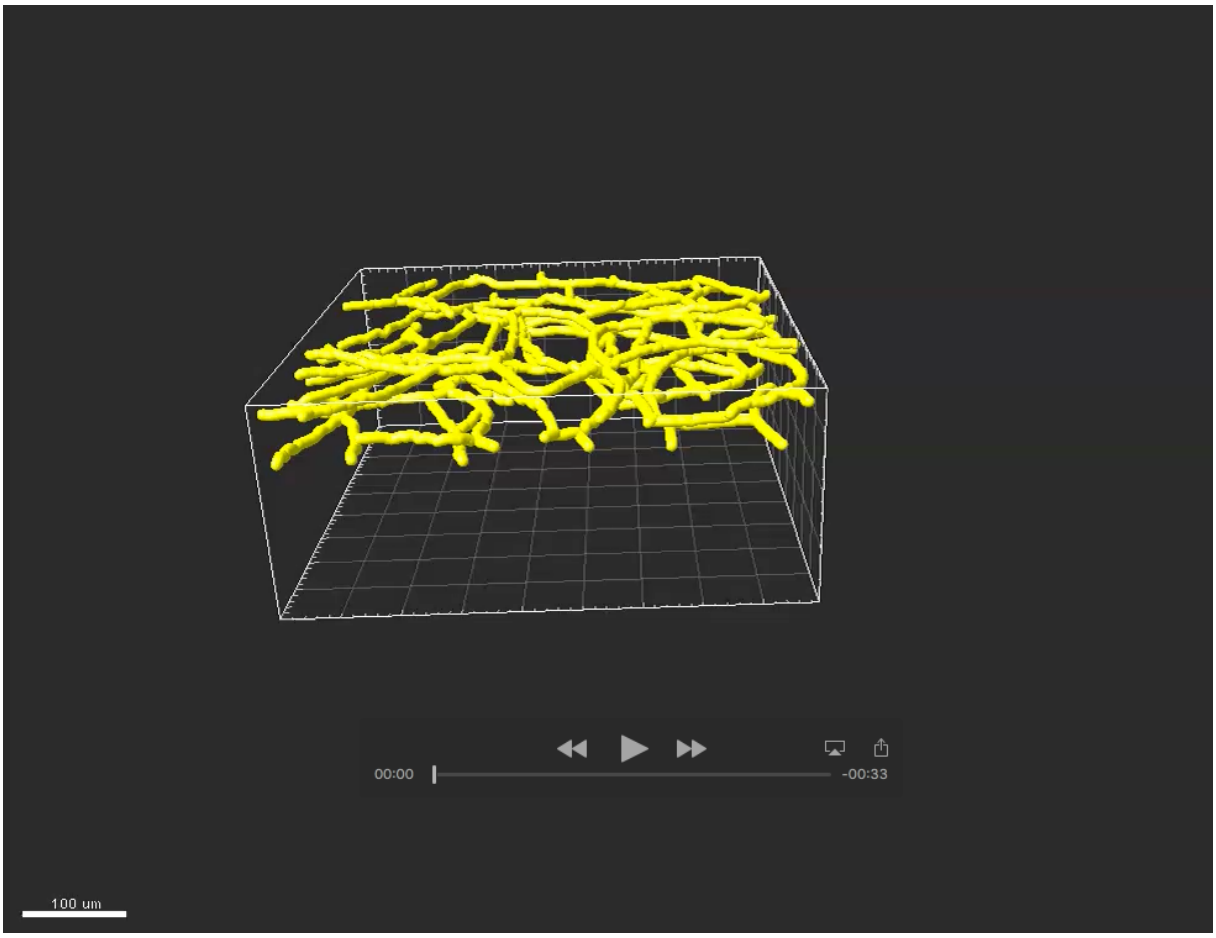

Supplemental movie 2:
Movie of the 3D reconstruction of multiphoton images obtained from constructs containing GFP-HUVECs and hASCs and Matrigel/rCOL. The calculated mean diameter is displayed. A multi-layered network is visible. Scale bar: 100 µm

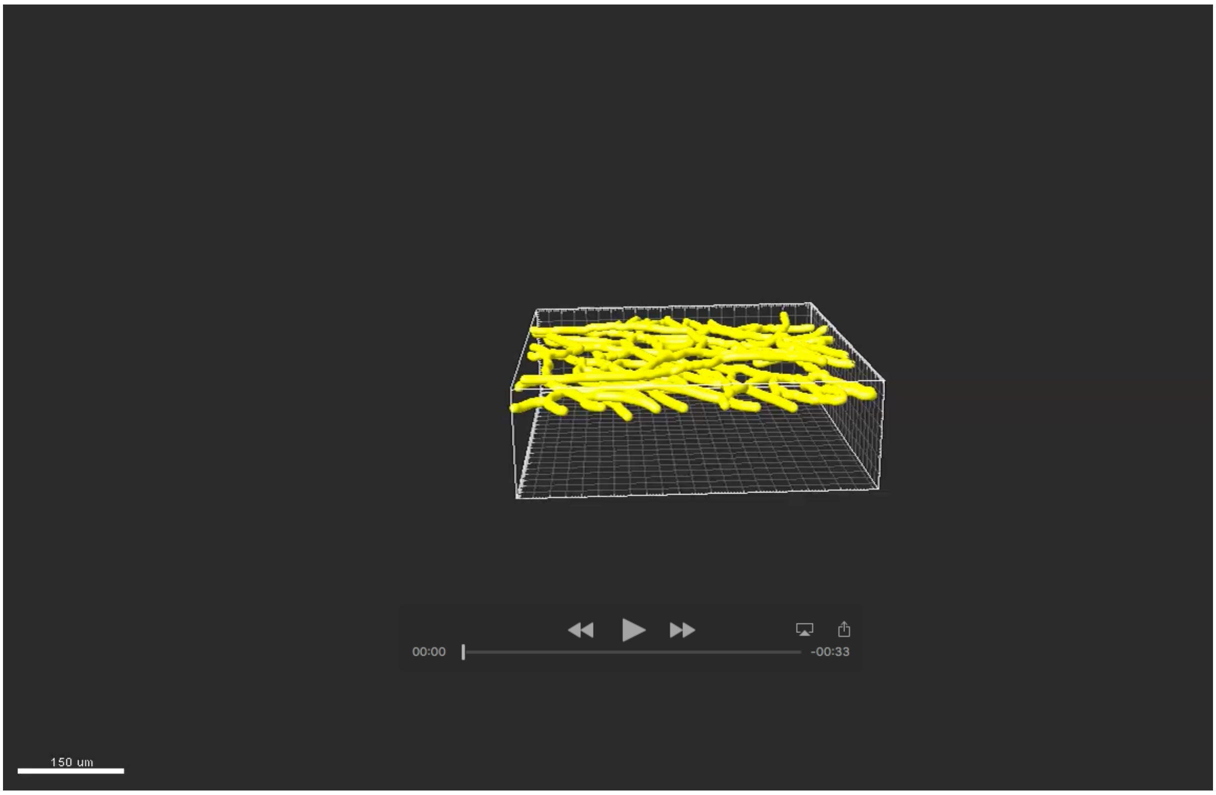

Supplemental movie 3:
Movie of the 3D reconstruction of multiphoton images obtained from constructs containing GFP-HUVECs and hASCs and hCOL. The calculated mean diameter is displayed. A multi-layered network is visible. Scale bar: 100 µm
